# Passive amylin immunotherapy improves brain function and reduces brain β‐amyloid pathology in APP/PS1 mice

**DOI:** 10.1002/alz70855_106792

**Published:** 2025-12-24

**Authors:** Deepak Kotiya, Florin Despa

**Affiliations:** ^1^ University of Kentucky, Lexington, KY, USA

## Abstract

**Background:**

Amylin, a systemic hormone that is co‐secreted with insulin by pancreatic β‐cell, co‐aggregates with brain parenchymal and vascular β‐amyloid in persons with Alzheimer's disease (AD). The present study sought to assess the safety and side effects during and after the treatment period of passive amylin immunotherapy in the APP/PS1 mouse model of AD pathology and APP/PS1 mice in which human amylin replaced the mouse amylin gene (HuAmy‐APP/PS1).

**Method:**

We generated a polyclonal anti‐amylin antibody (P2) in rabbits using the *N*‐terminal of amylin peptide as an immunogen. Five‐month‐old APP/PS1 males were randomly assigned into rabbit Ig‐G vehicle‐control and P2‐injected group (*n* = 4/group). P2 was injected at 10 mg/kg of body weight three times a week for six weeks. Mice were monitored for visible side effects and immunocomplex disease‐related symptoms. Six weeks later, we assessed brain function with the novel object recognition test, body weight and blood glucose(BG) levels. P2‐antibody level was measured in plasma, brain, and kidney tissues using an immunoassay. Using MSD‐ELISA, we conducted comparative immunochemical Aβ‐analyses of hippocampal tissues. Histological evaluations of the brain and pancreas with H&E and Prussian blue staining to assessed vascular degeneration, angiopathy, and microhemorrhages. This study is being expanded (*n* = 4/group, progressing to *n* = 10/group) to include HuAmy‐APP/PS1 mice.

**Result:**

APP/PS1 mice injected with P2‐amylin antibody had significantly high levels of P2 in plasma than vehicle‐treated mice (*p* <0.001). P2‐antibody was not detected in brain and kidney tissues. Histological analyses revealed no induction of microhemorrhages, with normal meninges and blood vessels in antibody‐injected group. Pancreatic tissue exhibited normal, healthy islets compared to controls. Prussian blue staining shows no increase in brain and pancreas microhemorrhages. P2‐injected mice showed an enhanced trend of recognition memory (*p* = 0.1121) along with a lower BG level (*p* = 0.2602) as compared to controls. Due to the small sample size, Aβ‐levels did not show significant differences. A similar trend of lower BG (*p* = 0.8027) and enhanced recognition memory (*p* = 0.1876) was observed in the antibody‐injected HuAmy‐APP/PS1 mice (*n* = 4/group).

**Conclusion:**

These findings suggest that passive amylin immunotherapy is well‐tolerated in APP/PS1 mice and does not induce microhemorrhages or other adverse effects, supporting its safety for further investigation in HuAmy‐APP/PS1 mice.

